# Genetic, Functional, and Immunological Study of ZnT8 in Diabetes

**DOI:** 10.1155/2019/1524905

**Published:** 2019-02-26

**Authors:** Qiong Huang, Jie Du, Chengfeng Merriman, Zhicheng Gong

**Affiliations:** ^1^Department of Pharmacy, National Clinical Research Center for Geriatric Disorders, Xiangya Hospital, Central South University, Changsha 410008, Hunan, China; ^2^Department of Physiology, Johns Hopkins School of Medicine, 725 North Wolfe Street, Baltimore, MD 21205, USA

## Abstract

Zinc level in the body is finely regulated to maintain cellular function. Dysregulation of zinc metabolism may induce a variety of diseases, e.g., diabetes. Zinc participates in insulin synthesis, storage, and secretion by functioning as a “cellular second messenger” in the insulin signaling pathway and glucose homeostasis. The highest zinc concentration is in the pancreas islets. Zinc accumulation in cell granules is manipulated by ZnT8, a zinc transporter expressed predominately in pancreatic *α* and *β* cells. A common ZnT8 gene (*SLC30A8*) polymorphism increases the risk of type 2 diabetes mellitus (T2DM), and rare mutations may present protective effects. In type 1 diabetes mellitus (T1DM), autoantibodies show specificity for binding two variants of ZnT8 (R or W at amino acid 325) dictated by a polymorphism in *SLC30A8*. In this review, we summarize the structure, feature, functions, and polymorphisms of ZnT8 along with its association with diabetes and explore future study directions.

## 1. Introduction

Zinc is an essential ion for the body and maintains important biological functions acting as a catalyst, regulator, or structural component [1]. Intracellular zinc concentration is finely tuned by many proteins to maintain cellular homeostasis. The metallothionein (MT) family of zinc-binding proteins as well as zinc importers, known as ZIPs or SLC39 proteins, control the intracellular zinc uptake, while zinc transporters, known as ZnTs or SLC30 proteins, control the efflux of zinc into the extracellular matrix or intracellular vesicle [2, 3]. Zinc functions as a “cellular secondary messenger” in the regulation of insulin signaling and glucose homeostasis [3], and zinc accumulation in granules is essential for insulin packaging, maturation, crystallization, trafficking, secretion, and regulation [4]. The concentration of zinc is about 30 mM in granules of the pancreatic islet *β* cells. Zinc is involved in the crystallization of insulin within secretory granules at pH 5.5 until its secretion [5]. Preproinsulin is synthesized by ribosomes as a single-chain molecule, and the signal peptide is removed immediately to generate proinsulin [6]. Proinsulin is folded in the endoplasmic reticulum and binds with zinc to form a hexamer by electrostatic coupling at the acidic granules [7]. As the granules mature in the Golgi apparatus, proinsulin undergoes proteolytic processing which mediated by prohormone convertase to dissociate the C-peptide [6]. Six proinsulin monomers bind with two central zinc ions at histidine 30 in the B-chain to form a hexamer resulting in crystallized proinsulin. In contrast to the granules, the free cytosolic zinc concentration is only about 0.4 nM [8], and it is important to maintain a high zinc gradient in the granules. If the zinc balance was disrupted, an abnormally high intracellular zinc concentration would cause cellular toxicity through thiol-dependent redox systems or by chelation of essential anions [4]. Diabetes is a metabolic disorder characterized by high blood glucose levels and decreased insulin secretion or increased insulin resistance [9, 10]. Ninety percent of diabetes is type 2 diabetes mellitus (T2DM) [11]. Pancreatic *β* cell dysfunction and/or insulin resistance are two major pathophysiological features of T2DM. Type 1 diabetes mellitus (T1DM) is an autoimmune disease which is caused by T cell-mediated destruction of the pancreatic *β* cells [12]. Significantly decreased serum zinc levels and increased urinary zinc loss are characteristics of both T1DM and T2DM patients [13–15]. Some studies have shown that zinc supplement or high dietary zinc intake can reduce the risk of T2DM [16], which is modified by obesity and genotype effects [17].

Zinc transporter protein member 8 (ZnT8) is thought to be the regulator of zinc concentration in *β* cells and also acts as a zinc sensor. ZnT8 is a unique protein as it is exclusively expressed in islets, and it facilitates the transport of zinc from the cytoplasm into secretory vesicles [18, 19]. ZnT8 is associated with both T1DM and T2DM, as an antigenic target and a mediator of zinc enrichment in insulin secretory granules [2], respectively. In T1DM, the presence of ZnT8 autoantibodies is used as an important diagnostic tool. Genome-wide association studies (GWAS) reported an association of ZnT8 gene (*SLC30A8*) mutation with T2DM [20]. The risk single nucleotide polymorphism (SNP) rs13266634 was correlated to T2DM disease susceptibility in several populations. More recent studies focus on ZnT8 function, expression, and distribution, but there are some controversial or conflicting results regarding its function. This paper will review previous results as well as our studies to elucidate the role of ZnT8, its association with diabetes, and its future study directions.

## 2. ZnT8 Features and Expression

**Figure 1:**
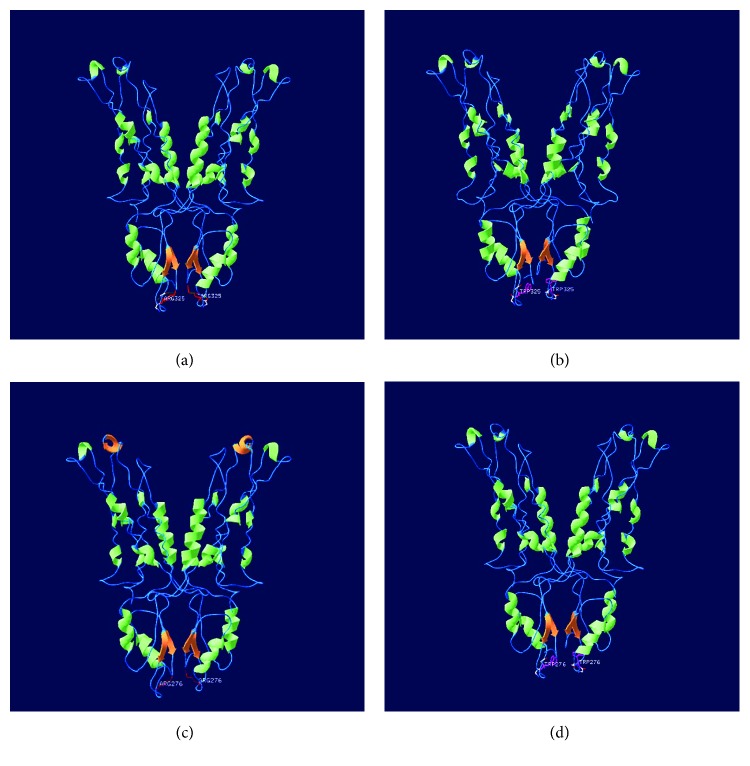
Homology model of the human ZnT8 homodimer with different forms and genotypes. The image in (a) is R325 in A form. The image in (b) is W325 in A form. The image in (c) is R325 in B form. The image in (d) is W325 in B form.

ZnT8 belongs to a subgroup of the cation diffusion facilitator (CDF) super family. It is a transmembrane protein composed of 369 amino acids in A form and 320 amino acids in B form, according to an N-terminal extension (Figure 1) [21]. Although the exact structure is not clear, ZnT8 is a dimer-membrane protein and conforms to a “Y”-shaped structure similar to immunoglobulin architecture according to the bacterial YiiP X-ray structure [22, 23]. Each monomer consists of six transmembrane domains (TMDs) and a histidine-rich loop with both the N- and C-terminals located in the cytoplasm [2]. ZnT8 acts as a Zn^2+^/H^+^ exchanger [2] and pumps zinc ions from the cytosol into intracellular vesicles or into the extracellular space [21]. The antiport mechanism could be induced by conformational changes in the protein structure. There are four zinc-binding sites located in each protomer. The primary zinc transport site resides at the TMDs (site A), and the secondary site is located in the interface between the membrane and the cytoplasmic domain (site B) [22, 23]. The C-terminal cytosolic domain has important functions in protein-protein interactions and as a zinc sensor [15].

ZnT8 is presented in a cell- or tissue-specific manner. It is mainly expressed in the pancreatic islets and localized to the membrane of insulin granules of *β* cells but can also be detected in pancreatic *α* cells [24]. At the mRNA level, ZnT8 is the zinc transporter with the highest expression in pancreatic tissues by far. At a level comparable to *β*-actin [25] or GAPDH [26], it has more than ten times the expression of other ZnT family members. This distribution pattern has been confirmed by a single-cell transcriptome study (RNA-Seq) of human pancreatic islets [27]. ZnT8 gene transcription is controlled by Pdx-1, a transcription factor enriched in *β* cells [28]. ZnT8 seems to be cytokine sensitive as its expression can be downregulated by cytokines such as IL-1*β* and IFN-*γ* [29]. In our previous localization studies, we used the stable expression of human ZnT8 in the INS-1E cell line to show specific antibody binding to the protein extracellular surface. We found abundant cell surface staining of ZnT8 and its coupling to glucose-stimulated insulin secretion (GSIS) which demonstrated the potential of ZnT8 as a biomarker for tracking and isolating functional *β* cells in mixed cell populations [30]. There have been many efforts to find *β* cell surface markers with cell sorting potential [31, 32], and ZnT8 may prove to be a useful marker. ZnT8 can act in imaging, isolation of live *β* cells from a heterogeneous population, or selection of differentiated functional *β* cells from progenitors [30].

## 3. ZnT8 Genetic Study in Human

ZnT8 genetic studies mainly focus on mutations in its coding gene *SLC30A8* which are associated with the risk of T2DM. The high-throughput GWAS assay provided the wild C allele variant of rs13266634 in *SLC30A8* associated with T2DM risk in French [33, 34], Finnish [34], and English populations [35, 36], and it has been replicated in other populations such as Asian [37], Chinese [38–44], Japanese [45–48], Singaporean [49], Korean [50, 51], Arab [52], Norwegian [53], Caucasian women [54], African-American [55], Pakistani [56], Tunisian [57], Mexican mestizo [58], Saudi Arabian [59, 60], Kazakh [61], Iranian [62], Mayan [63], Thai [64], and Greek-Cypriot [65]. The mutated T allelic frequencies in the Asian papulation were higher. Furthermore, the meta-analysis also reported that the rs13266634 polymorphism is among the most confirmed genetic markers for T2DM [66–74].

Not only is the SNP associated with T2DM, but there is also a relationship of rs13266634 with a substantial set of metabolic traits. Carriers with the rs13266634 C allele showed a significant decrease in insulin secretion [75–82] or a reduced proinsulin to insulin conversion [83]. The risk allele also shows a relationship with insulin sensitivity [84] and glucose tolerance from the Quebec Family Study [54]. The rs13266634 polymorphism showed a significant association with impaired glucose metabolism or impaired *β* cell function in Russian populations [75] and with HbA1c in Thai populations [64]. The risk C allele was correlated with lower acute insulin response to glucose, and it was also correlated with a lower disposition index in a Chinese study [82]. A common genetic risk C variant in *SLC30A8* influences, to different extents, the development of impaired fasting glucose (IFG), the transition from IFG to T2DM [77, 80, 85], and the impairment of glucose metabolism [75, 78].

The rs13266634 polymorphism is a nonsynonymous SNP which causes C to be substituted by T at base pair position 973 causing the amino acid to change from arginine (R) to tryptophan (W) at position 325 (Arg325Trp, R325W). The odds ratio (OR) value of the R form of ZnT8 for the risk of having T2DM is about 1.2. Other SNPs in *SLC30A8*, such as rs3802177 and rs11558471 [86] located in the 3′ untranslated region as well as rs16889462, which changes amino acid 325 from arginine (R) to glutamine (Q) (Arg325Gln, R325Q) [87], were also significantly associated with T2DM. Polymorphisms rs3802177 and rs11558471 are in strong linkage disequilibrium with rs13266634 [42, 88]. These genetic studies have shown that higher total zinc intake may attenuate the glucose-raising effect as well as lower fasting glucose levels [42] of the rs11558471 variant. SNP rs11558471 was strongly associated with T2DM (*P* = 0.002, OR = 1.334, 95% CI = 1.110 to 1.602) and moderately associated with diabetic nephropathy in a Malay population [89]. Sun et al. reported that rs11558471 was associated with higher circulating proinsulin and lower insulinogenic index [16].

Not all of the human studies can identify the link between the rs13266634 polymorphism and the risk of susceptibility to T2DM. Phani et al. [76] and Khan et al. [90] confirmed the association of rs13266634 with T2DM, but another two studies showed no detectable association [91, 92]. T2DM was not found in association with this polymorphism in studies reported among Mexican American Families [93] and in African Americans of European descent [94]. There are also some controversial study results in Indian populations. The inconsistencies may be due to racial differences, different sample selection criteria, or alternative assay detection methods. Another interesting result from Flannick et al. reported some loss-of-function mutations in the *SLC30A8* gene which could protect against the risk of T2DM [95]. These 12 rare protein-truncating variants were statistically associated with a 65% decrease in T2DM risk. The strongest protective association was observed in Iceland between p.Lys34SerfsX50 and measuring random glucose levels in nondiabetic subjects [95]. This interesting result implied that this allele could be a new target for antidiabetic drug therapy. Another nonsense variation, R138X (c.412 C>T), has shown significant association with T2DM (OR = 0.46, *P* = 0.012). Moreover, experiments with the recent *SLC30A8* R138X knock-in mouse model showed that insulin secretion was increased under hyperglycemic challenge, although the mouse had normal body weight, glucose tolerance, and *β* cell mass [26]. The phenotype could possibly be related to a decrease in mitochondrial gene expression and an increase in the expression of the voltage-gated proton channel Hv1 (Hvcn1) [26]. These data also provide evidences in favor of targeting ZnT8 as a possible therapy for T2DM.

## 4. ZnT8 Functional Studies *In Vivo* and *In Vitro*

How does ZnT8 expression level affect target cell functions? In the INS-1E cells, the consequences of ZnT8 overexpression were an increase in zinc accumulation and an enhancement of GSIS compared with control cells [26]. Cells with downregulated ZnT8 not only showed a functional decrease in insulin content and secretion in response to hyperglycemic stimulus but also showed fewer dense-core vesicles via electron microscopy [96]. These data suggest that ZnT8 is a key *β* cell functional protein for zinc accumulation and insulin secretion.

**Figure 2:**
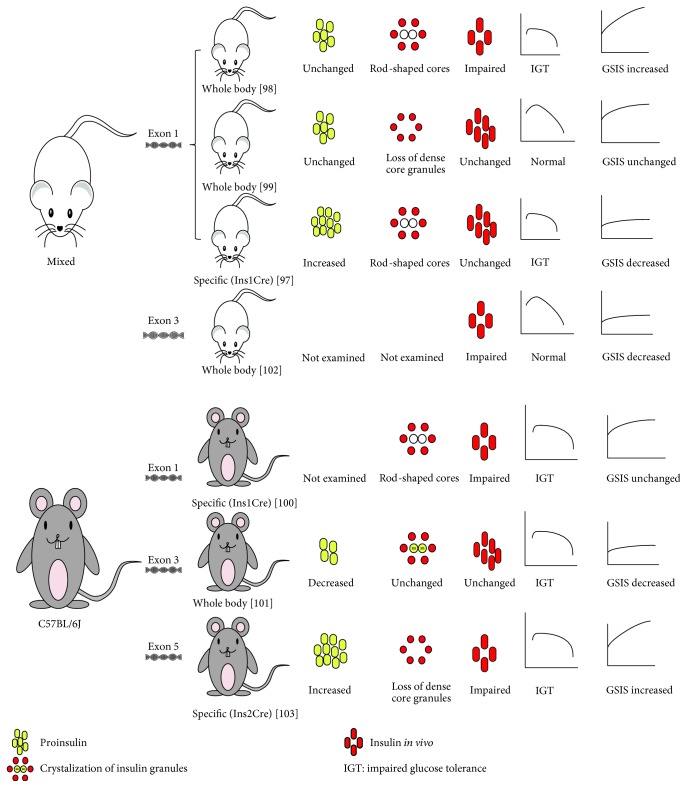
ZnT8 knockout mice in mixed genotypic mouse or C57BL/6J mouse showed a chaos phenotype.

Genetic mouse models used to investigate the whole animal or cell-specific ZnT8-KO effect on metabolic traits have confusing results. Exon 1 [97–100], exon 3 [101, 102], or exon 5 [103] were deleted in C57BL/6J [100, 101, 103, 104] or a mixed species mouse [97–99, 102] (Figure 2). All the ZnT8-KO animals showed no significant changes in growth, body weight, gross anatomy, or behavior compared with respective WT mice. Nearly all the ZnT8-KO mice showed no change in islet size, number, cell position, islet insulin content, and *α*/*β* cell ratio [98, 99, 101–103]. However, all of the studies showed that islet zinc content decreased. Some studies showed that the islet structure changed [97–100, 103], and pale insulin “progranules” were detected [97, 99], which implied that ZnT8 is necessary for zinc accumulation in islet cells. GSIS studies in the isolated islets showed no change [99, 100], a decrease [97, 101, 102], or an increase [98, 103]. During *in vivo* studies, the fasting glucose levels showed no change [97, 99, 101–103] or an increase [98]. The fasting insulin content also showed no change [97, 99, 101] or a decrease [98, 100, 102, 103]. The intraperitoneal glucose tolerance test (IPGTT) showed impaired glucose tolerance (IGT) [97, 98, 100, 101, 103] or normal glucose tolerance [99, 102]. Oral glucose tolerance test (OGTT) studies also showed controversial results from no change [101] to IGT [97]. Results from GSIS studies *in vivo* showed decreased [98, 103] or unchanged [97]. The variation of metabolic-related phenotypes in ZnT8-KO mice may result from using different methods and different animal genetic backgrounds. Another study using the combined deletion of *SLC30A7* and *SLC30A8* found that *SLC30A8* is critical for GSIS because a significant decrease of GSIS can be detected in both *SLC30A7/SLC30A8-*KO but not in the *SLC30A7* single-KO animal. These data unmask the function of ZnT8 in islets by the removal of ZnT7 and imply that ZnT8 may affect T2DM susceptibility through other tissues where it is expressed at low levels rather than through effects on pancreatic islet function [105]. In isolated human islets, the risk C allele does not affect ex vivo insulin secretion or *SLC30A8* expression. It also does not affect ZnT8 protein expression which is correlated with that of insulin and glucagon secretion [66].

Diabetes is a metabolic disease, although the environment plays an important role in its pathogenesis and development. One study used a *β* cell-specific ZnT8-KO mouse model and a whole ZnT8-KO mouse model with a high-fat, high-calorie diet to investigate the phenotype as measured by metabolic indicators. In the *β* cell-specific models, measurements indicated no weight change but a decrease of insulin synthesis and secretion was noted in addition to glucose intolerance. In the whole ZnT8-KO mouse model, after maintenance on a high-fat feed, the animals were significantly obese, hyperglycemic, hyperinsulinemic, insulin resistant, and glucose intolerant. The researchers concluded that ZnT8 is important to the whole body and not just in *β* cells only. The mechanism of diet-induced obesity in the ZnT8-KO mouse contributed to the T2DM risk in a *β* cell-specific and nonspecific manner [106]. More evidences for the role of ZnT8 in diabetes were determined by Tamaki et al., who found that ZnT8-KO mice had lower peripheral blood insulin levels, although they had hypersecretion of insulin from pancreatic *β* cells relative to WT animals. The secreted insulin was cleared by the liver during its first passage which was confirmed by the C-peptide/insulin ratio in the circulation [103]. This may help to explain the phenotype of ZnT8 SNP rs13266634 after knowing that the hepatic clearance of insulin may be impacted. These data imply that ZnT8 function should be evaluated *in vivo* as well as at the cellular and molecular levels.

In an *α* cell-specific ZnT8-KO mouse, the level of glucose and insulin tolerance was normal. However, the female models showed lower glucose infusion rates during hypoglycemic clamp experiments and increased glucagon secretion compared with WT controls. When responding to lower glucose (1 nM), islets isolated from *α* cell-specific ZnT8-KO mice secreted more glucagon than WT controls. This suggested that ZnT8 is important for glucagon secretion stimulated by lower glucose in a subset of *α* cells [24]. Alternatively, ZnT8 overexpression in *α* cells showed increased zinc accumulation in the cytoplasm and granules and impaired glucagon secretion in response to hypoglycemia [107]. These data provide further evidence that T2DM-associated polymorphisms in the *SLC30A8* gene may act in part via alterations in glucagon release and suggest that ZnT8 activation may restrict glucagon release in some settings.

## 5. Functional Mutation Studies

It has been difficult to confirm the functional phenotype attributed to the ZnT8 transporter polymorphism *in vivo*. Some studies have used purified human ZnT8 amino acid 325 protein to detect the function changes. Parsons et al. purified the cytoplasmic domain (CTD) of ZnT8 with R325 (ZnT8cR) or W325 (ZnT8cW) encoding mutations. The results showed that ZnT8cR was more thermostable than ZnT8cW, and the ZnT8cW monomers showed higher affinity to zinc [21]. A limitation of these studies is that they examined only the CTD instead of the full-length protein to evaluate ZnT8 activity. In our previous work, we used a highly sensitive functional assay *in vitro* to compare the functional difference between ZnT8's R form and W form. We found that the R form showed more activity when we induced expression in HEK293 cells, purified ZnT8 in lipid remodeling, and measured using the stop flow method [21]. These results showed a higher activity of the ZnT8 R form which confers increased risk of T2DM, whereas the ZnT8 W form displays lower functional activity. Thus, ZnT8 has the potential to be a new target for protection against T2DM.

## 6. ZnT8 and T1DMs

T1DM is an autoimmune disease characterized by T cell-mediated selective destruction of *β* cells in the pancreatic islets of Langerhans [108], the onset of which mainly occurs in childhood or young adulthood, and insulin is needed as an exogenous supplement. Autoantibodies including islet cytoplasmic autoantibodies (ICA), antibodies to insulin (IAA), the 65 kD form of glutamate decarboxylase (GADA), and the protein tyrosine phosphatase IA2 (IA2A) can be used to identify the onset of or risk of developing T1DM in more than 80% of the patients [108, 109].

ZnT8 is a novel islet autoantigen for T1DM, and it has been shown that ZnT8 was targeted by autoantibodies in 60–80% of new-onset T1DM patients, which occurred in only 2% of controls, 3% of T2DM patients, and 30% of patients with other autoimmune diseases associated with T1DM [108, 110]. If combined with the measurement of GADA, IA2A, and IAA, autoimmunity detection rates would rise to 98% at disease onset [111, 112]. It has also been confirmed that human ZnT8 autoantibodies showed no cross-reactivity with other ZnT family member proteins and no activation with mouse ZnT8 protein [113, 114]. IAA is usually the first autoantibody to appear at a young age in the preclinical stage compared to anti-ZnT8 autoantibodies, which are often not detected before 3 years of age [115, 116]. However anti-ZnT8 antibodies can detect up to 26% of T1DM patients who previously presented as autoantibody negative using IAA, ICA, or GADA assays [114]. Thus, anti-ZnT8 antibodies have been shown to exist in the blood from the prediabetic phase and are likely a downstream event of the primary genetic pathogenesis of T1DM [117]. It is a useful independent marker of autoimmunity either alone in antibody-negative subjects or combined with other T1DM autoantibody markers [115]. ZnT8 is a pancreatic-specific protein, and it could potentially be used to value the function of pancreatic islets.

ZnT8 is a multispanning transmembrane protein, and it is difficult to purify while maintaining its native structure. The existing antibody assays were preformed using radioimmunoprecipitation of the *in vitro* translation product from soluble CTD, N-domain, or fusion N- and C-terminal domains [108]. It is difficult to detect epitopes near transmembrane domains because no anti-TMD domain monoclonal antibody existed until now. Wan et al., through a combined bioengineering and nanotechnology approach, developed a proteoliposome-based full-length human ZnT8 antigen for efficient detection of a ZnT8 autoantibody on a plasmonic gold chip. This method showed efficient anti-ZnT8 antibody detection for T1DM diagnosis with about 76% sensitivity and 97% specificity [118]. The extra detection signal could come from autoantibodies targeting the extracellular surface(s) of ZnT8 in a pancreatic *β* cell. The specificity of ZnT8 autoantibody reactivity was influenced by SNP rs13266634 in the new proteoliposome-based assay. T1DM patients with different genotypes, namely, CC, CT, or TT, exert different phenotypic reactivities with respective ZnT8 antigen proteins. Of the patients with the CC genotype, 42% had the 325R antibody, 32% had 325R/325W dual antibodies, and only 5% had 325W-specific antibodies [119]. TT carriers showed that 73% had the 325R antibody, 13% had 325R/325W dual antibodies, and none had 325W-specific antibodies. 90% of the CT subjects showed a reaction with either 325R or 325W-specific antibodies and, recently, a subclass of serum anti-ZnT8 antibodies polyclone directed to the surface of live [120] and isolate-specific monoclonal anti-ZnT8 [121]. So ZnT8 is important for T1DM diagnosis, and its gene mutations also affect the autoantibody reactivity.

## 7. Summary

In this paper, we reviewed studies of the structure, expression, and function of ZnT8. Scientists appreciate that ZnT8 plays an important role in zinc accumulation in pancreatic islets. Controlling the zinc concentration is important not only in the intracellular compartment but also in its subcellular redistribution to vesicles. Previously, studies expressing ZnT8 in *β* cells demonstrated the substantial presence of ZnT8 and it has been difficult to distinguish the cytoplasmic versus the zinc granule distribution. Some studies focused on the *SLC30A8* gene mutations and the efficacy of antidiabetic drugs. We found that *SLC30A8* rs13266634 and rs16889462 polymorphisms were associated with the therapeutic efficacy of the antidiabetic drug repaglinide in Chinese T2DM patients [87]. In a diabetes prevention program study, 3007 subjects treated with metformin or troglitazone coupled with lifestyle changes for 1 year were genotyped and the results have shown that a genotype at *SLC30A8* could predict baseline proinsulin levels independently of insulin levels [122]. The *SLC30A8* rs13266634 (C allele) variant was associated with rosiglitazone therapy in HOMA-B, which is a homeostasis index assessing insulin secretion and *β* cell function. The C allele carriers showed a more active response to the drug [123].

ZnT8 has been confirmed to play an important role in maintaining glucose hemostasis. In human studies, carriers of the risk allele of *SLC30A8* have shown decreased insulin secretion, reduced proinsulin to insulin conversion, less insulin sensitivity, impaired glucose metabolism, and diminished *β* cell function. Results from animal studies were inconsistent, but these conflicting results may be due to inconsistent animal species, various KO methods, and/or different diet and other environmental conditions. Some important outcomes should be highlighted. Under normal condition, ZnT8 did not show a significant function phenotype, but it played an important role in the high-fat diet condition [106]. These show that gene and environmental interactions contribute to disease development as it is known in human T1DM. ZnT8 is highly sensitive to unbalanced glucose conditions, and both *α* and *β* cells contribute to glucose control in the whole body. Therefore, further investigation of ZnT8 function should be holistic and should include a broad panel of tests in response to the dynamic change of glucose. In our previous results, we found the ZnT8 risk mutation has increased functional activity, while loss-of-function mutations could protect from T2DM risk in humans; increased insulin secretion in animals was also reported. We showed that a membrane-embedded human ZnT8 antigen triggered a vigorous immune response in ZnT8-KO mice, and anti-ZnT8 autoantibodies which targeted the conformational (TM) epitopes could be beneficial to T1DM diagnosis. The conformational (TM) epitopes of ZnT8 are very important not only for detecting the cell surface ZnT8 expression, but also for function regulation by binding with cell surface ZnT8. Future studies are needed to clarify whether ZnT8 was blocked by anti-ZnT8 antibody effects on insulin secretion and whether it has a role for T2DM therapy.
